# Current Scenario of HIV/AIDS, Treatment Options, and Major Challenges with Compliance to Antiretroviral Therapy

**DOI:** 10.7759/cureus.515

**Published:** 2016-03-01

**Authors:** Adnan Bashir Bhatti, Muhammad Usman, Venkataramana Kandi

**Affiliations:** 1 Department of Medicine, Capital Development Authority Hospital, Islamabad, Pakistan; 2 Department of Medicine, Jinnah Hospital Lahore (JHL)/Allama Iqbal Medical College (AIMC), Lahore, Pakistan; 3 Department of Microbiology, Prathima Institute of Medical Sciences

**Keywords:** hiv, aids, drug adverse effects, hiv/aids, highly active antiretroviral therapy (haart), antiretroviral therapy

## Abstract

The discovery of the human immunodeficiency virus (HIV) as the causative organism of acquired immunodeficiency syndrome (AIDS) and the inability of modern medicine to find a cure for it has placed HIV as one of the most dreaded pathogens of the 21^st^ century. With millions of people infected with HIV, it was once thought to result in “medical apocalypse”. However, with the advent of antiretroviral therapy (ART), it is now possible to control HIV. Adherence to ART helps to keep the viral load under control and prolong the time of progression to AIDS, resulting in near normal life expectancy. Even with the introduction of ART, a substantial number of patients fail to adhere due to a variety of reasons, including adverse side effects, drug abuse, mental disorders, socioeconomic status, literacy, and social stigma. With the availability of so many options for HIV treatment at each stage of the disease progression, physicians can switch between the treatment regimens to avoid and/or minimize the adverse effects of drugs. Close monitoring, major social reforms, and adequate counselling should also be implemented to circumvent other challenges.

## Introduction and background

**Figure 1:**
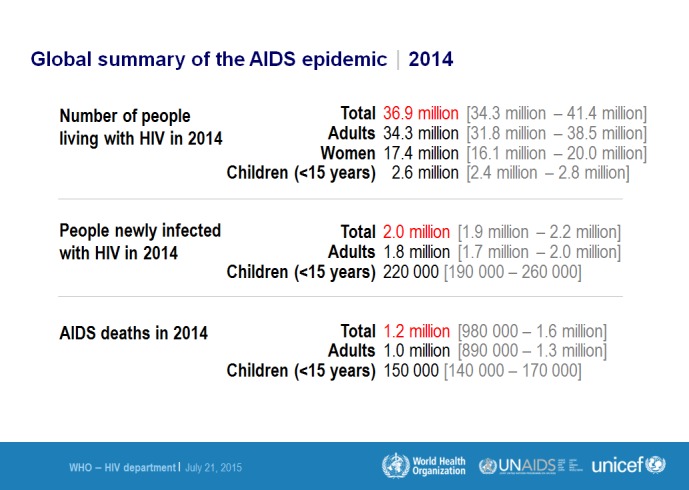
Prevalence of HIV/AIDS as of 2014. Figure retrieved from WHO website on December 6, 2015 from the link: http://www.who.int/hiv/data/epi_core_july2015.png?ua=1

Acquired immunodeficiency syndrome (AIDS) is a medical condition caused by the human immunodeficiency virus (HIV). HIV infection is a very current threat and can easily be termed as a curse upon the human race. The scientific community first noticed and recognized the presence of AIDS as an actual disease following an increase in the incidence of very rare opportunistic infections and cancers among otherwise healthy homosexual men [1]. HIV-1 was identified as the causative organism soon after the first official recognition of HIV patients in the USA [2]. HIV-2 was reported first in Africa in 1985 and is markedly different from HIV-1 [3]. It closely resembles a simian virus that infects macaques in captivity. Simian viruses that naturally infect African primates are suspected to reach humans via multiple cross-species transmissions resulting in the spread of HIV-1 and HIV-2 [2]. The global prevalence of HIV has expanded since its discovery and has now spread across the globe despite advances in antiretroviral treatments (ART). The mortality and morbidity rates related to HIV infections remain high in developing countries largely due to food insecurity and malnutrition [4]. Long-term concomitant sexual relationships and high infectivity during the early phase of HIV infections are other factors behind the extensive spread of HIV in the general population [5]. Figure 1 summarizes the number of victims by gender, incidence, and death, as well as the latest statistical data covering the 2014 AIDS epidemic [6]. 

## Review

### The infection

**Figure 2:**
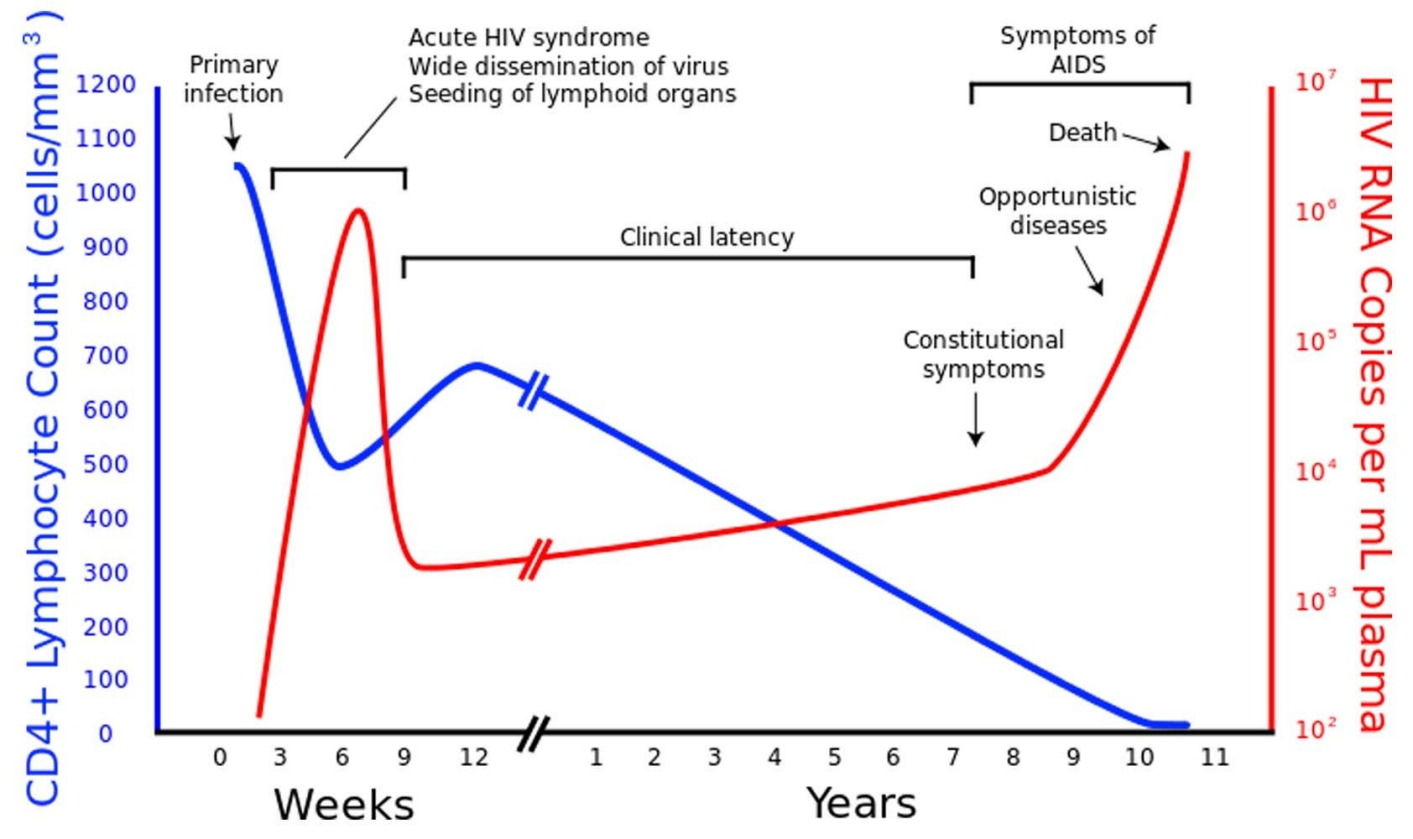
HIV time course. Figure retrieved from Wikipedia site on December 6, 2015 from the link: https://en.wikipedia.org/wiki/HIV#/media/File:Hiv-timecourse_copy.svg

The main site of the attack is the immune system, especially the CD4 T-lymphocytes (CD4 cells). Once infected, the virus gradually and silently overpowers the host’s defense mechanisms, resulting in opportunistic infections and cancers that are otherwise rare. Activated and differentiated CD4 cells have a pivotal role in the activation of cell-mediated and humoral immune systems [7]. HIV infection results in the depletion of CD4 cells in the peripheral blood [8]. Among untreated patients, the depletion continues over a course of several years until the patient succumbs to AIDS. It is the last stage of the HIV infection, and it presents itself anywhere between two and 15 years post-infection [9]. The following figure represents the timeline of HIV infection from the initial infection to the expression of AIDS-defining symptoms (Figure 2) [10].

### HIV subgroups

HIV -1

HIV-1 is well-known for its extensive genetic diversity. There are four different lineages coming under HIV-1: M, N, O, and P. The most commonly reported HIV virus across the globe is group M [2]. Group N less prevalent, reported only from Cameroon [11]. Group O is accountable for 1% of the total HIV-1 cases and is mainly found Cameroon and Gabon [12]. Group P is the rarest of all and has been identified in Cameroonian pregnant woman in France [13]. It has a prevalence of 0.06% of total HIV infections [14].

HIV-2

HIV-2 is most commonly reported in West Africa, with Guinea-Bissau and Senegal having the highest incidence. Eight different types of HIV-2 exist, labeled HIV-A to HIV-H. Group A is reported throughout the sub-Saharan region [15]. Group B is reported more commonly in the Ivory Coast [16]. Due to the sporadic nature of the infection and incidence, C to H are categorized as “dead-end” transmissions that produce no subsequent infections [2].

### Current status of HIV infection and mortality rate

Western, Central Europe, and North America

Approximately 2.4 million individuals are HIV-positive in this region. An estimated 85,000 new HIV infections were reported in 2014, and more than 50% of infections were from the United States of America. About 26,000 AIDS-related deaths were also reported in the same period [17].

Asia and Pacific

As of 2014, approximately five million individuals were previously infected in Asia and the Pacific, with as many as 340,000 new HIV infections arising that year. China, Indonesia, and India contribute to about 78% of the total new disease burden in Asia and the Pacific with about 240,000 deaths. Patients receiving ART are approximately 36%, with 3.2 million active HIV patients having no access to ART [17]. 

Pakistan

In Pakistan, the index case of HIV infections was reported in 1987 [18].^ ^As per the annual report of Pakistan National AIDS Control Program, the incidence of HIV has been increasing since first reported. According to UNAIDS, the joint United Nations program on HIV/AIDS, the total number of individuals with an active HIV infection is approximately 94,000. The prevalence rate among adults is between < 0.1% and 0.2%. Currently, there are as many as 26,000 women, age 15 and older, and approximately 2,100 children, up to age 14, currently living with HIV. The total number of AIDS-related deaths in this region was 2,800 in the year 2014 [19]. 

### Treatments options for HIV

**Figure 3:**
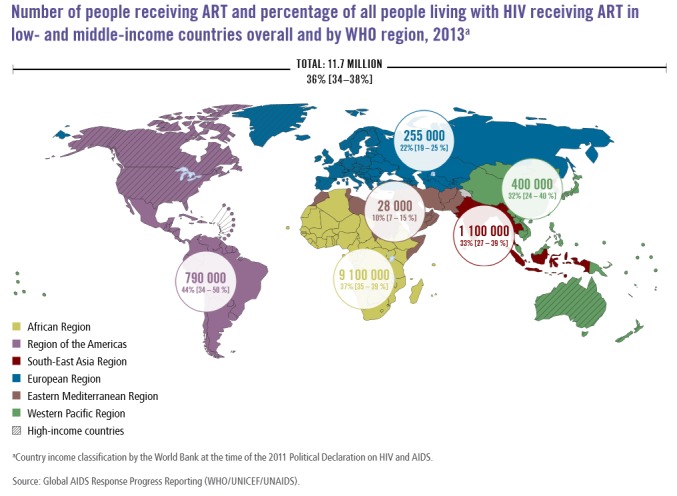
Percentage of HIV patients under antiretroviral therapy (WHO 2014). Figure retrieved from WHO website on December 6, 2015 from the link: http://www.who.int/hiv/data/artmap2014.png?ua=1

HIV infection has a very complex pathogenesis and varies substantially in different patients. Therefore, it can easily be considered as a very host-specific infection. The specificity of pathogenesis often complicates treatment options that are currently available for HIV infection [20].^ ^Effective management of HIV infection is possible using different combinations of available drugs. This method of treatment is collectively known as antiretroviral therapy (ART). Standard ART is comprised of a concoction of at least three medicines (termed as “highly active antiretroviral therapy” or HAART) [21]. Effective ART often helps control the multiplication of HIV in infected patients and increases the count of CD4 cells, thus, prolonging the asymptomatic phase of infection, slowing the progression of the disease, and also helps in reducing the risk of transmission. Figure 3 demonstrates the percentage of HIV patients under ART [22].

### FDA-approved HIV drug classes

Reverse Transcriptase Inhibitors 

Reverse transcriptase inhibitors are a group of drugs, which can bind and inhibit the reverse transcriptase enzyme to intercept the multiplication of HIV. There are two types of inhibitors: non-nucleoside reverse transcriptase inhibitors (NNRTIs) [23] and nucleoside reverse transcriptase inhibitors (NRTI) [24]. Examples of this group of drugs include zidovudine, didanosine, abacavir, tenofovir, and Combivir.

Protease Inhibitor

Regulation of HIV protease is of high importance for the correct assembly and production of HIV. Protease inhibitors effectively block the functioning of protease enzymes in acutely and chronically HIV-infected CD4 cells. Inhibition of HIV protease enzymes results in the liberation of immature and noninfectious viral particles [25]. Examples of this group of drugs include lopinavir/ritonavir, indinavir, ritonavir, nelfinavir, and amprenavir.

Fusion Inhibitors

This class of drugs acts by blocking HIV from entering the CD4 cells of infected patients. They inhibit the fusion of HIV particles with the CD4 cells [26]. Enfuvirtide is an example of a fusion inhibitor used in HIV treatment.

Chemokine Receptor 5 Antagonist

This group of drugs prevents the infection by blocking the chemokine receptor 5 (CCR5) antagonist receptor present on CD4 cells. In the absence of vacant CCR5 receptors, HIV fails to gain entry and infect the cell [27]. Maraviroc is an example of a CCR5 antagonist used in HIV treatment.

Integrase Strand Transfer Inhibitors 

Strand transfer inhibitors prevent the integration of viral DNA into the host genome of CD4 cells by an integrase enzyme. Blocking integrase prevents HIV from replicating [28].^ ^Raltegravir, elvitegravir, and dolutegravir are some medications in this category.

### Treatment regimen for HIV

Present HIV treatment guidelines recommend ART treatment for all patients, irrespective of the CD4 cell count, to improve and prolong the progression of disease to AIDS [29]. Adherence to treatment is of paramount importance in order to achieve the full efficacy of treatment and also to prevent the incidence of drug resistance [30].^ ^

### Latest WHO recommendations for ART

A concise form of first, second, and third line treatment options recommended by the World Health Organization (WHO) is given below [29].

First-line ART

Adults: First-line ART treatment for adults consists of two NRTIs and one NNRTI. Tenofovir disoproxil fumarate (TDF) + lamivudine (3TC) or emtricitabine (FTC) + efavirenz (EFV) as a fixed dose is the favored choice for this type of ART. When this drug combination is contraindicated or is unavailable, 1) zidovudine (AZT) + 3TC + EFV, 2) AZT + 3TC + nevirapine (NVP), or 3) TDF + 3TC (or FTC) + NVP is used.

Contraindications:

1. Creatinine clearance is less than 50 ml per minute: Tenofovir. 2. Patients on psychoactive drug treatment: Efavirenz. 3. Patients who are pregnant or who are trying to conceive: Efavirenz. 4. ALT elevation: Nevirapine.

Pregnant and breastfeeding patients: First-line ART in this subpopulation is comprised of a single daily dose of TDF + 3TC (or FTC) + NVP. Breastfeeding infants of mothers who are receiving ART must receive six weeks of infant prophylaxis with a daily dose of NVP. The preventive medication should commence immediately post-delivery or when HIV exposure is identified.

Pediatric patients: Patients below three years of age should be given Lopinavir/Ritonavir (LPV/r)-based treatment, even under NNRTI exposure. When LPV/r is not a viable option, NVP-based treatment should be used. For infected children who are over age three, EFV is the ideal NNRTI while NVP has been identified as the second option. For infected children younger than three years of age, who develop TB while on the Lopinavir/Ritonavir (LPV/r)-based treatment, the NRTI regimen should be switched to abacavir (ABC) + 3TC or AZT + 3TC until the TB infection is cleared. NRTI regimens similar to that of adults (TDF + 3TC (or FTC)) or (AZT + 3TC) or (ABC + 3TC) are preferred for patients between 10 and 19 years of age who weigh 35 kg or more.

Second-line ART

Adults, including pregnant and breastfeeding patients: When a first-line treatment of ART fails, a second-line ART should be utilized. The second-line ART is comprised primarily of two NRTIs and a ritonavir-boosted PI. The recommended option for second-line ART includes AZT and 3TC as the NRTI. After the failure of AZT or stavudine (d4T) + 3TC-based first-line regimen, TDF + 3TC (or FTC) as the NRTI should be considered. When first-line NNRTI-based treatment fails, two NRTIs + a boosted PI are suggested

Pediatric patients: For children below three years of age, first-line ART is continued even when it fails. No change in treatment is recommended; instead, adequate steps should be taken to improve adherence to the ART regimen. If first-line ART fails in children ages three and up, a second-line treatment consisting of one NNRTI and two NRTIs should be given. If ABC or TDF + 3TC (or FTC) fails, the recommended option is AZT + 3TC. After a failure of AZT or d4T + 3TC (or FTC) in first-line treatment, the preferred NRTI option is ABC or TDF + 3TC (or FTC).

Third-line ART

If first- and second-line ART fails, the WHO recommends inclusion of new medicines with the least amount of risk for development of cross-resistance towards previously used drugs (e.g. integrase inhibitors and second-generation NNRTIs and PIs).

### Factors to consider when selecting ART

The major factors that deserve thorough consideration while choosing an ART for a patient include the viral load and CD4 cell count before the treatment, the result of HIV genotypic drug resistance test, HLA-B*5701 status, patient preferences, and anticipated adherence. Comorbid conditions to screen prior to ART include cardiovascular disease, hyperlipidemia, renal disease, osteoporosis, psychiatric illness, neurologic disease, drug abuse or dependency requiring narcotic replacement therapy, pregnancy, coinfections with hepatitis C (HCV), hepatitis B (HBV), and tuberculosis (TB) [31].

### CD4 count monitoring for therapeutic response

Monitoring patients’ viral load is critical to identify ART response (WHO 2015). When the viral load analysis is not practical via polymerase chain reaction (PCR), branched chained DNA (bDNA), and nucleic acid sequence-based amplification (NASBA), the CD4 count is used as an indicator of HIV treatment response. During the first year of treatment, increases in CD4 count from 50 to 150 cells/mm^3^ with an increased response in the first trimester are considered as a positive response. CD4 count rises steadily ranging from 50 to 100 cells/mm^3^ per year until equilibrium is reached in the subsequent years (normal range: 500 cells/mm3 to 1200 cells/mm3) [32]. Periodic monitoring of CD4 count is required during and even after the patient achieves normal CD4 count under ART. A number of treatment independent factors like age, viral load, genetic make-up, lifestyle, quality of health care, etc., negatively influence the CD4 counts and HIV disease progression. Under such circumstances, a change in ART medication might be required.

### Major factors for ART non-adherence

Adverse Effects of ART

One of the major challenges that patients and physicians face with ART is the incidence of adverse drug reactions (ADR). ADR is defined as “a response to a drug that is noxious and unintended and occurs at doses normally used in man for the prophylaxis, diagnosis, or therapy of disease, or for modification of physiological function” [33]. ADR often persuades patients from continuing treatment, thus resulting in suboptimal efficacy. A serious consequence of treatment discontinuation is the emergence of drug resistance, making future therapeutic interventions ineffective [30]. 

The major adverse effects of ART can be grouped into the following categories:

1. Gastrointestinal: Nausea, diarrhea, vomiting, taste perversion, constipation, dyspepsia, abdominal pain, hepatotoxicity, and pancreatitis [34-35]. 2. Central nervous system: Headache, vision problems, dizziness, tinnitus, insomnia, paresthesia, pain/numbness/tingling in extremities, peripheral neuropathy, somnolence, excessive sleep at night, memory problems, loss of olfactory function, and hearing impairment [34]. 3. Hematological: Anemia, bilirubinemia, increased urate, and blood in the urine [35]. 4. Psychological: Anxiety, confusion, depression, nightmares, elation, and delusions [35].^^ 5. Metabolic: Abnormal fat distribution (lipodystrophy), anorexia, dyspnea, fatigue, lethargy, and weight gain [34-35]. 6. Dermatological: Skin rash, facial discoloration, and pruritus [35].^^ 7. Musculoskeletal*:* Body aches and vague chest pain [34].^^ 8. Miscellaneous: Hypersensitive reactions, oral ulcerations, fever, and irregular menstrual cycles [34].

Drug Abuse

Continuous drug abuse is an important risk factor in HIV/AIDS patients’ ART, nonadherence, and mortality [36]. In a study conducted on HIV-positive drug addicts in Canada, heroin and cocaine injections were reported to adversely affect adherence to ART [37].^ ^In a separate six-month long longitudinal study, which examined the effect of drug use and abuse on ART among 150 HIV positive patients, it was discovered that acute effects of intoxication negatively influence ART adherence. The major mechanisms by which drug abuse results in ART nonadherence include drug abuse induced neurocognitive/psychosocial impairment and psychiatric dysfunctions [38]. 

Mental Disorders

The prevalence of psychiatric disorders is reported to be very high among HIV-infected individuals [36]. In a longitudinal study investigating the mental health, substance abuse, and psychosocial predictors among HIV-positive mothers, the presence of psychiatric disorders, stressful lifestyles, suboptimal living conditions, and parenting stress were associated significantly with ART nonadherence [39]. Childhood sexual violence-induced anxiety and depression may also result in ART nonadherence [40]. Hazardous drinking is another significant precipitator of anxiety and depression among HIV patients that results in ART nonadherence [41]. 

Socioeconomic Status

Socioeconomic status is strongly associated with HIV-related mortality in the contemporary universal healthcare system because opportunities for patients of lower socioeconomic status to receive ART are meager. In a study conducted among HIV-positive Cambodian women, 80% of those who discontinued ART were of low socioeconomic status. The estimated risk for low adherence in this population was reported to be five times higher for women than those in a medium or high social position [42]. Poverty-induced stress is an important aspect that has to be addressed in issues regarding ART nonadherence [43]. The quality of housing and access to food are the two most important factors that prevent the poverty-ridden population from ART adherence [43].

Poor Literacy

Literacy is another major factor closely associated with ART nonadherence with people of lower health literacy experiencing higher illness severity than people with better health literacy [44]. Health literacy has been defined by the WHO as “the cognitive and social skills which determine the motivation and ability of individuals to gain access to, understand, and use information in ways which promote and maintain good health” [45]. Many reports suggested that the inability to comprehend medication instructions by illiterate HIV-positive patients is an important factor resulting in failure to follow accurate daily medication therapy [46].

Social Stigma

The stigma of HIV and AIDS is assumed to have a negative influence on ART adherence [47]. Stigma can be defined as an “attribute that is deeply discrediting” imposed by society that reduces someone “from a whole and usual person to a tainted, discounted one” [48]. In a cohort study conducted in five African countries (Lesotho, Malawi, South Africa, Swaziland, and Tanzania) among 1,457 HIV-positive patients over a period of 12 months, individuals perceiving a high HIV stigma reported greater nonadherence to ART. Symptom intensity is also high when compared to those who did not experience such a stigma [49]. One study conducted in South Africa reported that internalized stigma is responsible for 4.8% of the variance in cognitive-affective depression leading to ART nonadherence. Furthermore, the researchers urge the medical community to introduce social reform efforts to reduce stigma and assist people living with HIV/AIDS in adjusting and adapting [50]. 

## Conclusions

Recent advances in HIV treatments have dramatically altered the nature and progression of HIV/AIDS. It can be safely considered as a “chronic” disease, provided the infected patients receive proper ART. Unfortunately, current statistics of the worldwide HIV burden tells another story: one with a steady rate of HIV-related deaths. More people die of complications and the progression of HIV to AIDS than should be when ART is used properly. The major hurdle a physician faces with ART is the incidence of adverse side effects of the treatment, which persuade patients to discontinue the treatment. Poverty, lack of awareness, and the social stigma associated with the infection complicate an already complicated situation. Appropriate changes in treatment regimens and medications can help patients overcome such adverse effects and potential complications inherent to the disease. Additionally, it is highly advisable to provide patients and their immediate family members with appropriate counseling for treatment compliance and psychological support. 
